# Lysine-specific demethylase 1 (LSD1) regulates macrophage plasticity and immune checkpoint expression in colorectal liver metastases

**DOI:** 10.1186/s43556-026-00602-5

**Published:** 2026-10-20

**Authors:** Candela Cives-Losada, Cristiana Soldani, Michela Anna Polidoro, Barbara Franceschini, Flavio Milana, Aurora Gazzillo, Antonella Cammarota, Chiara Camisaschi, Francesca Clemente, Achille Anselmo, Luca Di Tommaso, Guido Torzilli, Domenico Mavilio, Ana Lleo, Matteo Donadon

**Affiliations:** 1 Department of Health Sciences, Università del Piemonte Orientale, Novara, Italy; 2 Laboratory of Hepatobiliary Immunopathology, IRCCS Humanitas Research Hospital, Rozzano, Milan Italy; 3 Department of Biomedical Sciences, Humanitas University, Pieve Emanuele, Milan, Italy; 4 Division of Hepatobiliary and General Surgery, Department of Surgery, IRCCS Humanitas Research Hospital, Rozzano, Milan Italy; 5 Flow Cytometry Core, IRCCS Humanitas Research Hospital, Rozzano, Milan Italy; 6 Spectral Flow Unit, Fondazione Istituto Nazionale Di Genetica Molecolare-INGM, Milan, Italy; 7 Department of Pathology, IRCCS Humanitas Research Hospital, Rozzano, Milan Italy; 8 Unit of Clinical and Experimental Immunology, IRCCS Humanitas Research Hospital, Rozzano, Milan Italy; 9 Department of Medical Biotechnology and Translational Medicine, University of Milan, Milan, Italy; 10 Division of Internal Medicine and Hepatology, Department of Gastroenterology, IRCCS Humanitas Research Hospital, Rozzano, Milan Italy; 11 Division of General Surgery, University Maggiore Hospital Della Carità, Novara, Italy

**Keywords:** Liver metastasis, Tumour microenvironment, Tumour immunology, Tumour-associated macrophages

## Abstract

Colorectal liver metastases (CRLM) are a major cause of mortality in colorectal cancer and are characterised by a profoundly immunosuppressive tumour microenvironment in which tumour-associated macrophages (TAMs) play a central role. However, the molecular mechanisms driving TAM plasticity and maintenance remain poorly understood. Here, we investigated macrophage heterogeneity in CRLM and examined the contribution of the epigenetic regulator lysine-specific demethylase 1 (LSD1) to TAM polarisation. Analysis of patient-derived specimens by flow cytometry, immunohistochemistry and transcriptional profiling identified a population of large TAMs (L-TAMs) enriched in the peritumoral niche that displayed an immunosuppressive, M2-like phenotype and was associated with poor clinical outcome. In contrast, smaller TAMs (S-TAMs) exhibited a more inflammatory transcriptional programme, including increased IL1β expression. Tumour-conditioned media reproduced these distinct macrophage phenotypes in vitro, indicating that tumour-derived factors actively shape TAM identity. Pharmacological inhibition of LSD1 using baicalin or INCB059872 reduced the expression of CD206 and the immune checkpoint molecule PD-L1, consistent with macrophage reprogramming away from a tumour-promoting state. Collectively, these findings identify LSD1 as an epigenetic regulator of macrophage plasticity in CRLM, implicated in the maintenance of immunosuppressive TAM phenotypes, and potentially able to induce phenotypic reversion. Our findings identify LSD1 as a regulator of TAM phenotype and support further investigation of LSD1 targeting as a strategy to remodel the metastatic tumour microenvironment and potentially enhance anti-tumour immunity.

**Supplementary Information:**

The online version contains supplementary material available at https://doi.org/10.1186/s43556-026-00602-5.

## Introduction

Colorectal cancer (CRC) represents a significant burden of cancer mortality worldwide, with the liver representing the most common organ harbouring distant metastasis. Colorectal liver metastases (CRLM) are associated with poor prognosis and limited therapeutic options [1]. The tumour microenvironment (TME) in metastatic CRC is highly immunosuppressive, limiting the efficacy of systemic therapies, including immune checkpoint blockade. The crosstalk between tumour cells and their microenvironment is essential for tumour initiation, growth, and progression [2], while the continuous remodelling of the TME further promotes tumour evolution and immune evasion [3].

Among the immune populations within the TME, tumour-associated macrophages (TAMs) are key regulators of tumour growth, angiogenesis, and immune suppression [2]. TAMs can arise from distinct sources: long-lived tissue-resident macrophages (derived from yolk sac–progenitors) [4] or circulating monocytes (derived from bone marrow haematopoietic stem cells) that infiltrated tissues and subsequently differentiate into TAMs [5, 6]. Their remarkable plasticity allows them to acquire different phenotypes depending on the characteristics of the surrounding microenvironment, ranging from pro-inflammatory M1-like macrophages with anti-tumour functions to M2-like macrophages exhibiting a pro-tumour phenotype [7]. Although this M1/M2 framework does not fully capture the complexity of macrophage states in vivo, it remains a useful conceptual model to describe the functional spectrum of TAM activation. Soluble factors such as cytokines are critical drivers of this phenotypic plasticity, orchestrating the local immune response [8].

In CRLM, the morphological features of TAMs within the peritumoral region have been correlated with patient prognosis. Large TAMs (L-TAMs) have been associated with adverse clinical outcomes, suggesting that macrophage morphology reflects functionally distinct states with prognostic relevance in CRLM [9]. Unlike transcriptomic classifications, macrophage morphology can be readily assessed in routine histopathological specimens, making it an attractive surrogate marker to investigate macrophage heterogeneity and its clinical relevance. This morphological and functional heterogeneity underscores the pivotal role of TAMs as key orchestrators of tumour progression, modulating immune responses and shaping the metastatic TME [10]. It also identifies the tumour margin as a dynamic interface where cellular interactions and cytokine gradients govern macrophage polarisation and promote immune escape, suggesting that modulation of TAM plasticity may represent a promising therapeutic strategy [11].

Among emerging approaches, epigenetic modulation—which involves heritable changes in gene function without altering the DNA sequence of a gene [12]—has gained attention as a mean to re-educate TAMs toward a pro-inflammatory, anti-tumoral phenotype. Epigenetic regulators are increasingly recognized as mediators of macrophage differentiation and functional plasticity, making them attractive therapeutic targets within the TME [13]. In this context, lysine-specific demethylase 1 (LSD1, *KDM1A*), an epigenetic regulator of myeloid cell differentiation and function, controls gene expression by modifying histone marks [14]. Beyond its established role in tumour cells, LSD1 regulates immune cell identity and macrophage plasticity and is highly expressed in immunosuppressive TAM subsets, particularly L-TAMs, in CRLM [15]. However, whether LSD1 contributes to the maintenance of immunosuppressive phenotypes and immune checkpoint regulation in the metastatic niche remains unclear. LSD1 can be pharmacologically targeted by compounds such as baicalin, a natural flavonoid with anti-inflammatory properties derived from plants of the *Scutellaria genus*, and INCB059872, a potent irreversible LSD1 inhibitor currently under clinical evaluation [16].

In this study, we combined the phenotypic characterisation of patient-derived TAMs with functional in vitro macrophage models to investigate how epigenetic regulation contributes to macrophage heterogeneity in CRLM. We show that L-TAM enrichment is associated with poor clinical outcome and that pharmacological inhibition of LSD1 attenuates immunosuppressive macrophage features, including CD206 and PD-L1 expression. Collectively, our findings identify LSD1 as a regulator of macrophage plasticity and support epigenetic modulation as a potential strategy to remodel the metastatic immune microenvironment.

## Results

### Characterisation of macrophage phenotypes in healthy and peritumoral tissue from patients with CRLM

Liver tissue from patients with CRLM (*n* = 14) was collected during surgical resection, one liver sample distant from the tumour, which was considered as healthy tissue, and one fragment of tissue adjacent to the tumour, which was defined as peritumoral, were obtained. The samples were then processed, stained with the indicated antibodies and analysed by FACS.

**Fig. 1:**
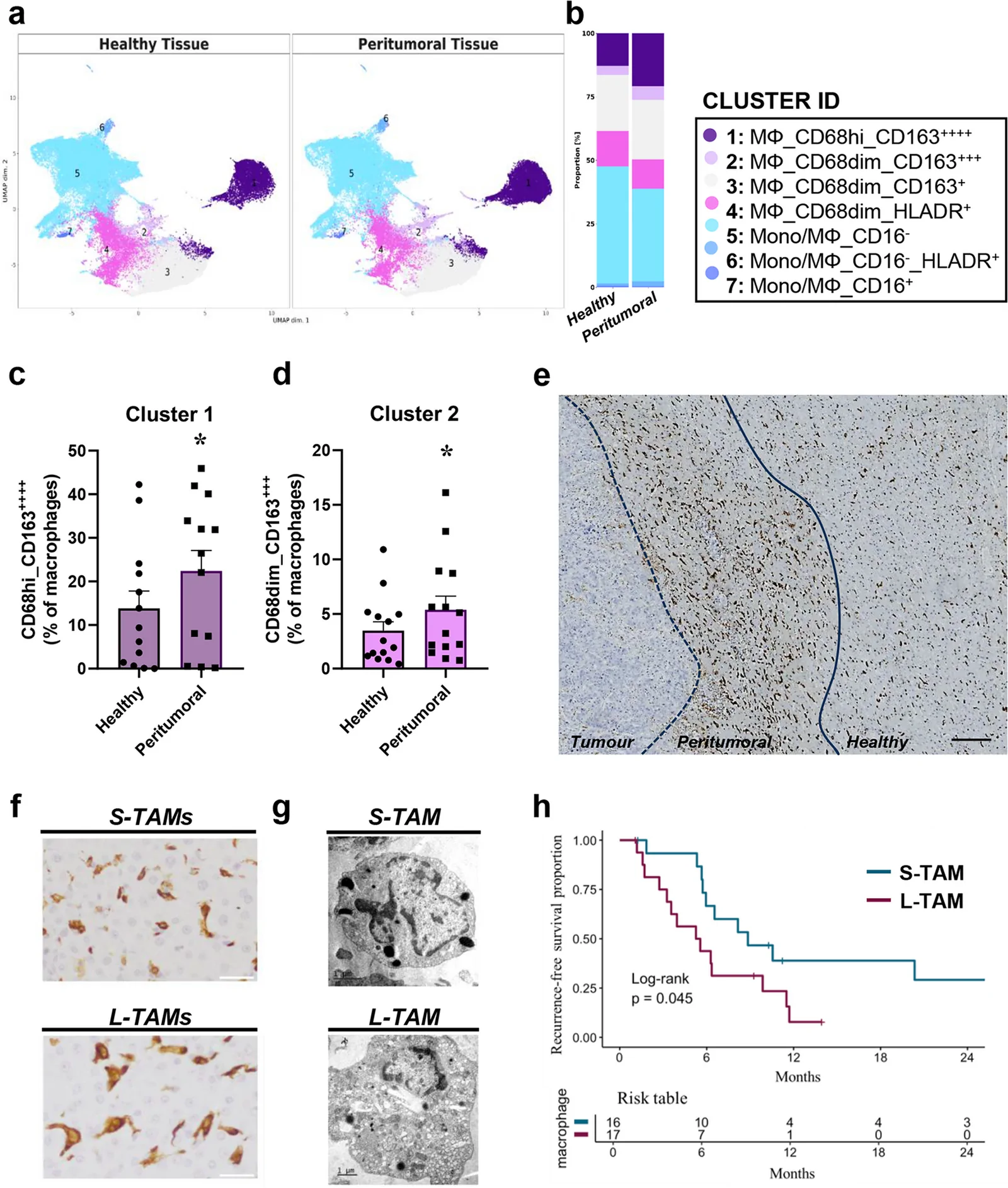
Characterisation of monocytes/macrophages populations in paired healthy and peritumoral liver tissues from colorectal liver metastases (CRLM). **a** UMAP plots illustrating the distribution of cell clusters based on the expression of flow cytometry markers in healthy versus peritumoral samples (*n* = 14), and **b** bar graphs depict the relative proportion of each cluster within total macrophages. Percentage of cells of **c** cluster 1 (CD68hi_CD163^++++^) and of **d** cluster 2 (CD68dim_CD163.^+++^) within the total macrophages isolated from CRLM samples (healthy or peritumoral). Values are mean ± SEM. *, *p* < 0.05 as compared with the macrophages isolated from healthy tissue (**c**,**d**). **e** Immunohistochemistry (IHC) staining for CD163 highlights TAMs (20X). **f** Representative images illustrate distinct TAM morphologies in the peritumoral area, with patients showing a predominance of either small TAMs (S-TAMs) or large TAMs (L-TAMs) (scale bar, 30 µm). **g** Transmission electron microscopy images displaying macrophages with diverse morphologies (scale bar, 1 µm). **h** Two-year recurrence-free survival of 33 patients resected for CRLM stratified based on TAM morphology (*n* = 16 for S-TAMs abundance and *n* = 17 L-TAMs abundance) in the peritumoral region (*p* = 0.045)

This flow cytometry analysis allowed for the identification of distinct macrophage subpopulations. Once the macrophages were distinguished (CD45^+^/CD33^+^/CD123^−^/CD15^−^/CD11b^+^/CD68^+^) (Fig. S1), an unsupervised analysis was performed, revealing seven different clusters (Fig. 1a). Clusters 1–4 displayed a macrophage-like phenotype, characterized by higher CD68 compared with clusters 5–7 [5, 17, 18] which retained a more monocytic phenotype, with higher levels of CD14 (clusters 5 and 6) or CD16 (cluster 7) (Fig. S2a). Interestingly, among the mature macrophages we observed a high heterogeneity as defined by the variable expression of CD68 and HLA-DR, CD163 and CD206 polarisation markers. It is worth noting that macrophages with high levels of CD68 (CD68-hi; cluster 1) constituted a highly homogeneous cell subset which showed high levels of CD163, CD206, HLA-DR (Fig. S2b) and forward scatter (FSC) (Fig. S2c) signal intensity. The cells in cluster 1 exhibited the features of L-TAM we have previously described in the peritumoral area of CRLM [9]. By contrast, mature macrophages with low levels of CD68—defined as CD68-dim—showed smaller dimension (Fig. S2c) than L-TAM and were further subdivided in three different clusters according to CD163 expression (distinguishing cluster 2 and 3) and HLA-DR (high in cluster 4), the latter being more M1-like (Fig. S2d and Fig. 1a and b). These data do not exclude a monocyte origin of S-TAMs; rather, they indicate that, at the time of analysis, S-TAM-associated macrophage clusters are phenotypically distinct from the less differentiated monocytic populations concurrently detected within the same tissue.

After defining the different mono/macrophage subsets, we compared their distribution between healthy and peritumoral tissues in our patient cohort, thus observing a significantly higher frequency of cells in clusters 1 (CD68hi_CD163^++++^) and 2 (CD68dim_CD163^+++^) within the total macrophage population in the peritumoral area (Fig. 1c and d).

Interestingly, macrophage infiltration was greater in the peritumoral zone compared to healthy compartments, as demonstrated by CD163-stained tissue (Fig. 1e). Notably, consistent with our previous findings, the peritumoral area contained heterogeneous macrophage populations with distinct sizes and morphologies, classified as S-TAMs and L-TAMs (Fig. 1f) [9, 19]. Following tissue enzymatic digestion and sorting of peritumoral macrophages based on size and CD163 expression, the cells were processed for electron microscopy to assess morphological differences. This analysis revealed that S-TAMs are smaller, with a more regular and rounded morphology, whereas L-TAMs exhibited increased cellular size, irregular shape and greater intracellular complexity, including prominent intracellular vacuolar structures (Fig. 1g).

Based on the immunohistochemistry (IHC) characterization of macrophages, survival analysis showed that after a median follow-up of 13.9 months (95% CI: 10.3–33.6), patients with increased L-TAMs (high CD68⁺/CD163⁺ TAMs) had a reduced median recurrence-free survival (RFS) (5.4 months vs. 8.8 months, *p* = 0.045, Fig. 1h). Notably, this occurred despite no differences in baseline disease burden (number or size of CLRM) or response to chemotherapy (Table 1 and Table S1), further validating previously reported data [9, 19].

**Table 1:** Comparison of CRLM patients’ clinical characteristics according to macrophage size (Small vs Large)

Variable	Small macrophages (*n* = 16)	Large macrophages (*n* = 17)	*p*
***Age,**** (Median [IQR])*	66.13 [52.74, 72.00]	58.24 [48.76, 71.52]	0.494
***Sex, male,**** n (%)s*	10 (62.5)	11 (64.7)	1.000
***BMI**** (Median [IQR])*	24.53 [21.40, 26.41]	24.22 [21.72, 27.13]	0.666
***Site of primary tumour,**** n (%)*			0.585
*Right*	4 (25.0)	4 (23.5)	
*Transverse*	1 (6.2)	2 (11.8)	
*Left*	4 (25.0)	7 (41.2)	
*Rectum*	7 (43.8)	4 (23.5)	
***Grade stage 3,**** n (%)*	2 (20.0)	5 (45.5)	0.440
***Tumour stage,**** n (%)*			0.536
*1–2*	1 (6.2)	2 (11.8)	
*3–4*	15 (93.8)	15 (88.2)	
***Nodal stage,**** n (%)*			0.286
*0*	2 (12.5)	3 (17.6)	
*1*	10 (62.5)	6 (35.3)	
*2*	4 (25.0)	8 (47.1)	
***Neoadjuvant CHT, yes,**** n (%)*	13 (81.2)	17 (100.0)	0.205
***Response to CHT***			0.960
*CR*	0/13 (0.0)	0/17 (0.0)	
*PR*	10/13 (76.9)	13/17 (76.5)	
*SD*	2/13 (15.4)	3/17 (17.6)	
*PD*	1/13 (7.7)	1/17 (5.9)	
***Extrahepatic disease, yes,**** n (%)*	2 (12.5)	3 (17.6)	1.000
***Synchronous disease, yes,**** n (%)*	16 (100.0)	15 (88.2)	0.493
***Bilobar disease, yes,**** n (%)*	12 (75.0)	14 (82.4)	0.928
***BRAF mutated, yes,**** n (%)*	15 (100.0)	17 (100.0)	1
***KRAS mutated, yes,**** n (%)*	3 (18.8)	7 (41.2)	0.307
***CEA**** (Median [IQR])*	4.60 [3.08, 9.03]	3.90 [2.40, 5.10]	0.732
***CA19-9**** (Median [IQR])*	11.05 [3.80, 18.48]	15.80 [7.10, 63.10]	0.154
***Number of tumour**** (Median [IQR])*	5.50 [3.75, 7.75]	5.00 [2.00, 11.00]	0.731
***Size of largest tumour**** (Median [IQR])*	2.95 [1.45, 4.12]	3.50 [2.50, 4.10]	0.416
***Adjuvant CHT, yes,**** n (%)*	8 (50.0)	13 (76.5)	0.223

*BMI* body-mass index, *CHT* chemotherapy, *CR* complete response, *PR* partial response, *SD* stable disease, *PD* progressive disease, *BRAF* B-Raf proto-oncogene, *KRAS* Kirsten rat sarcoma virus oncogene homolog, *CEA* carcinoembryonic antigen

### Transcriptional profiling reveals distinct functional programmes in S-TAMs and L-TAMs from human CRLM

**Fig. 2:**
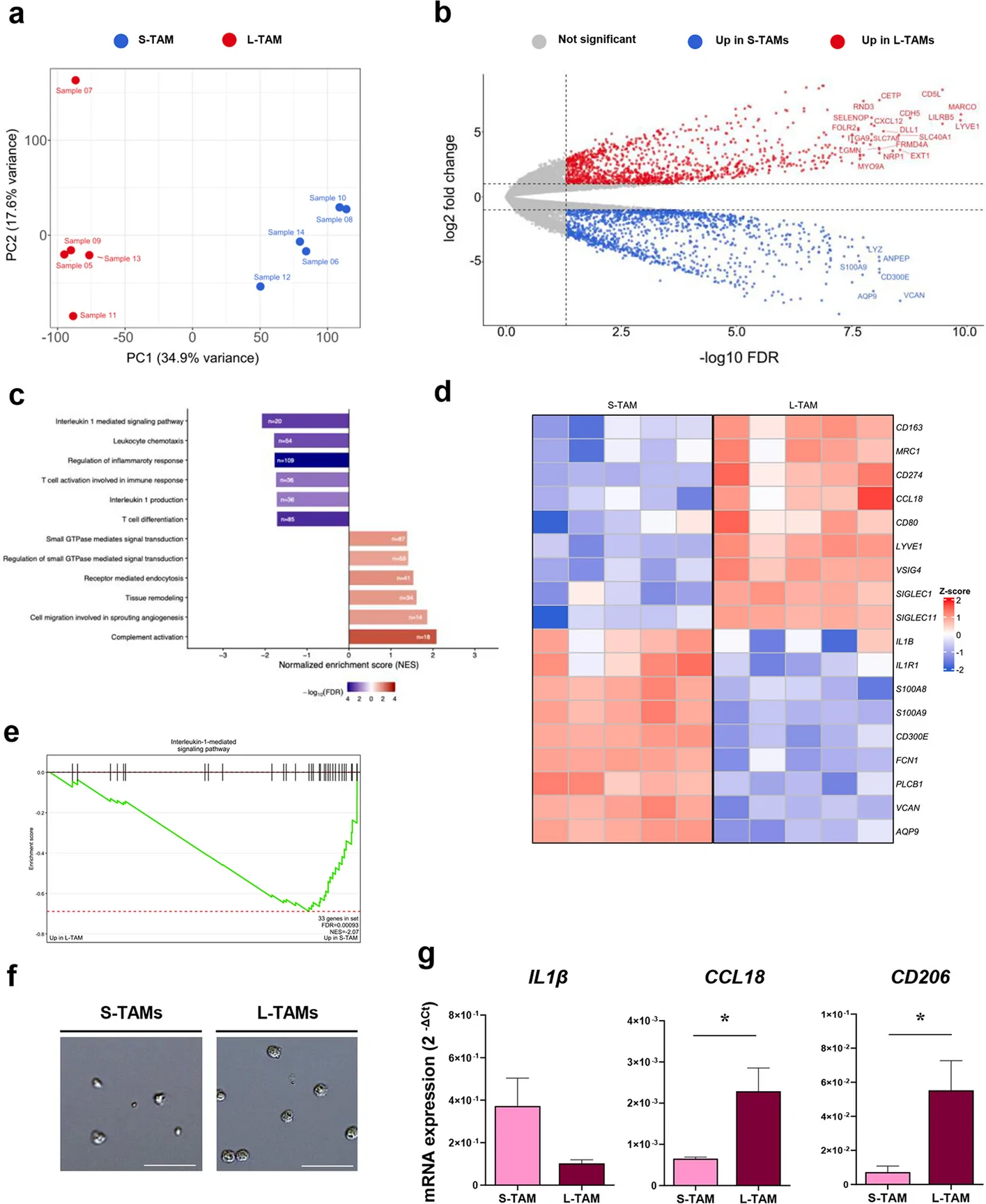
Transcriptomic profiling of small (S-) and large (L-) tumour-associated macrophages (TAM) from colorectal liver metastases (CRLM). **a** Principal component analysis (PCA) of bulk RNA-sequencing data from paired sorted S- and L-TAM isolated from the peritumoral region of CRLM patients (*n* = 5). **b** Volcano plot showing differentially expressed genes (DEGs) between L-TAMs and S-TAMs. **c** Gene set enrichment analysis of large and small tumour-associated macrophages. Bar plot showing significantly enriched Gene Ontology Biological Process pathways in L-TAMs (positive NES) and S-TAMs (negative NES). Bar length represents the normalised enrichment score (NES), and colour intensity indicates the adjusted p value. **d** Heatmap showing row-scaled expression of representative DEGs defining the L-TAM and S-TAM transcriptional signatures. Values represent gene-wise Z-scores calculated from log2-transformed TMM-normalised expression values. **e** GSEA running enrichment score for the GO Biological Process interleukin-1-mediated signalling pathway. Genes were ranked from those most highly expressed in L-TAMs to those most highly expressed in S-TAMs. **f** Bright-field microscopy images of cultured FACS-sorted TAMs (scale bar, 50 µm). **g** Gene expression of selected genes in FACS-sorted TAMs from CRLM, as assessed by RT-qPCR*. GAPDH* was used as normalizer for sorted TAMs. Values are mean ± SEM (*n* = 3 at least per subset). *, *p* < 0.05; as compared with S-TAMs

To further characterise the biological features associated with morphologically defined TAM populations, we reanalysed previously generated bulk RNA-sequencing data from paired sorted S-TAM and L-TAM populations isolated from the peritumoral tissue of CRLM patients (*n* = 5) [9]. These macrophage populations had been previously identified based on their morphological features and CD163 expression; here, we performed an integrative transcriptomic analysis to define the molecular programmes associated with each TAM state (Fig. 2).

Principal component analysis demonstrated a clear separation between S-TAM and L-TAM paired samples, indicating distinct transcriptional profiles associated with these morphologically defined macrophage populations (Fig. 2a). Differential expression analysis identified multiple differentially expressed genes (DEGs) between L-TAMs and S-TAMs (Fig. 2b). L-TAMs displayed enrichment of transcriptional programmes associated with immune regulation, tissue remodelling and scavenger functions, whereas S-TAMs showed increased expression of genes associated with inflammatory responses.

Functional enrichment analysis of differentially expressed genes revealed distinct biological programmes associated with each macrophage population. S-TAMs were significantly enriched in pathways associated with inflammatory and immune activation, including regulation of inflammatory response, interleukin-1-mediated signalling, interleukin-1 production, leukocyte chemotaxis, T-cell differentiation and activation involved in immune responses (Fig. 2c). These findings indicate that S-TAMs preferentially exhibit inflammatory-associated transcriptional programmes and may contribute to the recruitment and activation of immune cells within the peritumoral microenvironment. In contrast, L-TAMs were enriched in biological processes related to complement activation, receptor-mediated endocytosis, tissue remodelling, angiogenesis, and small GTPase-mediated signalling (Fig. 2c), consistent with macrophage functions involved in tissue adaptation, matrix remodelling, phagocytic activity, and tumour-supportive responses rather than inflammatory activation.

To define representative molecular signatures of each morphologically defined TAM population, we selected a panel of DEGs representative of the enriched biological programmes. Consistent with the pathway analysis, L-TAMs preferentially expressed genes associated with tissue-resident and immunoregulatory macrophage functions, including *CD163, MRC1 (CD206), CD274, CCL18, LYVE1, VSIG4,* and *SIGLEC1*, whereas S-TAMs showed increased expression of inflammatory-associated genes, including *IL1B, IL1R1, S100A8, S100A9, FCN1, CD300E, VCAN*, and *AQP9* (Fig. 2d). Moreover, the enrichment of the interleukin-1-mediated signalling pathway in S-TAMs was further illustrated by GSEA running enrichment plots (Fig. 2e). Together, these data demonstrate that the previously described morphologically defined L-TAMs and S-TAMs are underpinned by distinct functional transcriptional programmes rather than differences in morphology alone.

To validate these transcriptional signatures, peritumoral macrophages were FACS-sorted from an independent cohort of patients with CRLM (*n* = 4) according to their morphology and CD163 expression, as previously described. Following cell sorting, macrophages were either cultured for morphological assessment (Fig. 2f) or processed for RT-qPCR analysis (Fig. 2g). Consistent with the transcriptomic analysis, S-TAMs displayed higher expression of the inflammatory-associated marker *IL1β*, whereas L-TAMs expressed significantly higher levels of the immunoregulatory markers *CCL18* and *CD206* (Fig. 2g), confirming the distinct molecular programmes associated with these morphologically defined macrophage populations.

### Generation of S-TAM-like and L-TAM-like macrophage models using tumour cell-derived conditioned media

To investigate whether tumour-derived soluble factors contribute to the establishment of distinct TAM phenotypes, we generated in vitro macrophage models resembling the S-TAM and L-TAM states identified in CRLM patient samples. Conditioned media were obtained from patient-derived tumour cell cultures established from surgically resected CRLM specimens (Fig. S3a). Tumour samples were stratified according to the predominant TAM morphology observed by IHC in the peritumoral area, identifying S-TAM-enriched and L-TAM-enriched tumours.

**Fig. 3:**
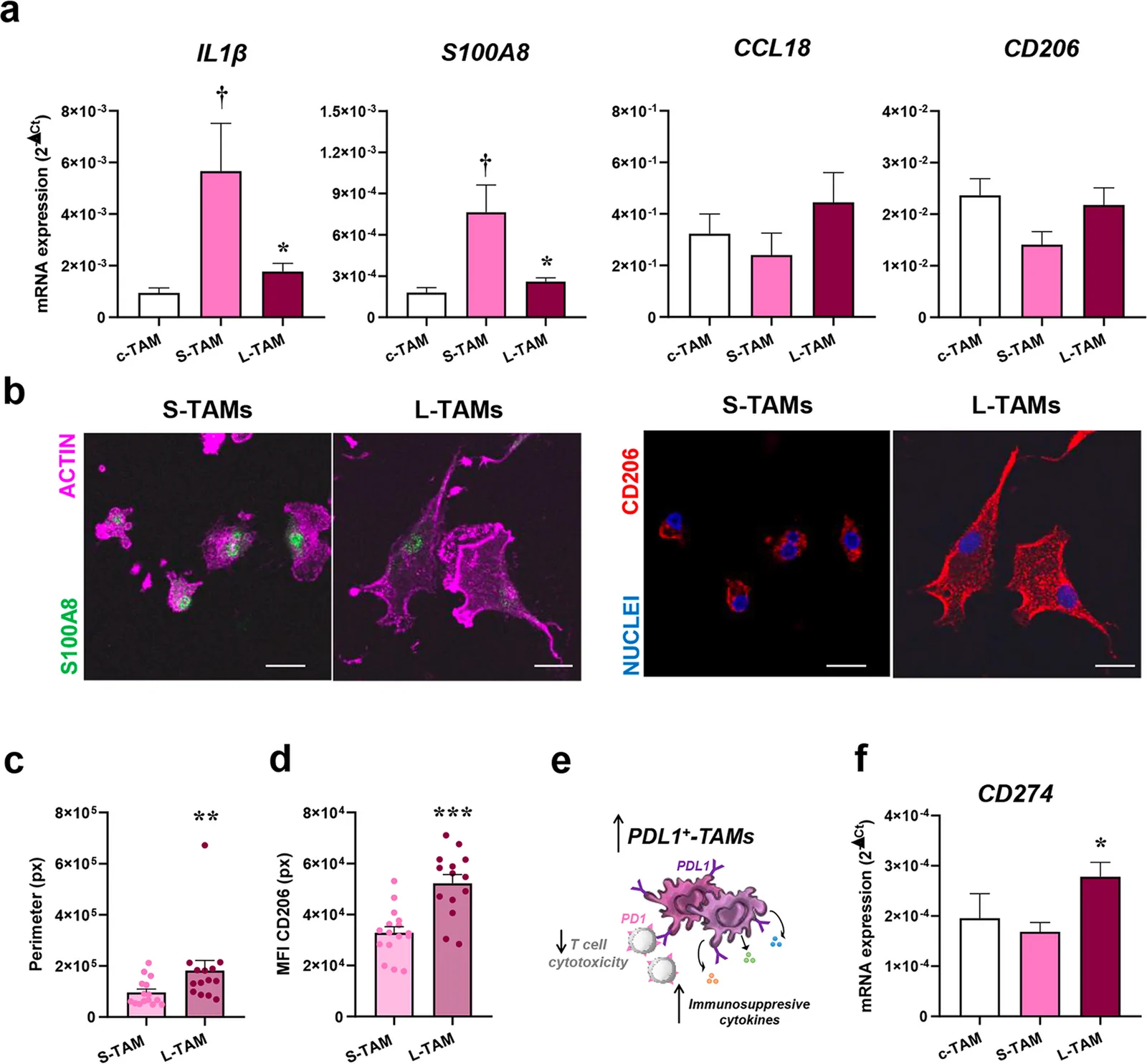
Tumour-conditioned media from CRLM with distinct small (S-) and large (L-) tumour-associated macrophages (TAM) abundance induces divergent macrophage phenotypes in vitro. **a** Gene expression of selected genes in in vitro-generated TAMs, as assessed by RT-qPCR*.* **b** Immunofluorescence staining of S100A8 (green) and actin filaments (with phalloidin in magenta) or CD206 (red) and nuclei (with Hoechst, blue) in in vitro-derived S-TAMs and L-TAMs (scale bar, 10 µm). **c** Quantification of cell perimeter and **d** CD206 mean fluorescence intensity (MFI). **e** Schematic representation of the mechanisms by which PD-L1 expression in TAMs promotes immune evasion and suppression of anti-tumour T-cell responses. **f** *CD274* (coding for PD-L1) expression was evaluated in in vitro-generated TAMs. Double normalisation with *GAPDH* and *18S* was used. Values are mean ± SEM (*n* = 3 at least per subset). *, *p* < 0.05; **, *p* < 0.01; ***, *p* < 0.001; as compared with S-TAMs or **†**, *p* < 0.05, as compared with classical TAMs (c-TAMs)

Exposure of macrophages to tumour cell-derived conditioned media induced distinct phenotypic states consistent with the characteristics observed in patient-derived TAM subsets. Conditioned media from L-TAM-enriched tumours promoted macrophage features associated with the L-TAM phenotype, whereas conditioned media from S-TAM-enriched tumours induced a more inflammatory macrophage profile. Gene expression analysis showed that macrophages exposed to L-TAM-enriched tumour supernatants displayed increased expression of genes associated with immunoregulatory macrophage activation, while S-TAM-like macrophages exhibited enhanced expression of inflammatory macrophage-associated genes (Fig. 3a).

Consistent with these transcriptional differences, immunofluorescence analysis revealed distinct morphological and phenotypic features between the two macrophage states. S-TAM-like macrophages showed a smaller, more rounded morphology and reduced expression of CD206, whereas L-TAM-like macrophages exhibited a larger and more irregular morphology together with increased CD206 expression (Fig. 3b-d).

To explore the immunomodulatory properties associated with these TAM subsets, we analysed the expression of *CD274*, encoding the immune checkpoint ligand PD-L1 (Fig. 3e), which emerged among the differentially expressed genes identified by RNA sequencing between sorted S-TAMs and L-TAMs from CRLM patient samples (Fig. 2d). Consistently, validation in our in vitro model showed increased PD-L1 expression in macrophages exposed to conditioned media derived from tumour cells isolated from L-TAM-enriched tumours compared with those exposed to conditioned media from S-TAM-enriched tumours (Fig. 3f).

### Cytokines released by patient-derived CRLM cells

To identify soluble factors potentially involved in shaping macrophage phenotypes, we performed a cytokine profiling of media collected from patient-derived tumour cell cultures established from surgically resected CRLM samples (*n* = 24).

**Fig. 4:**
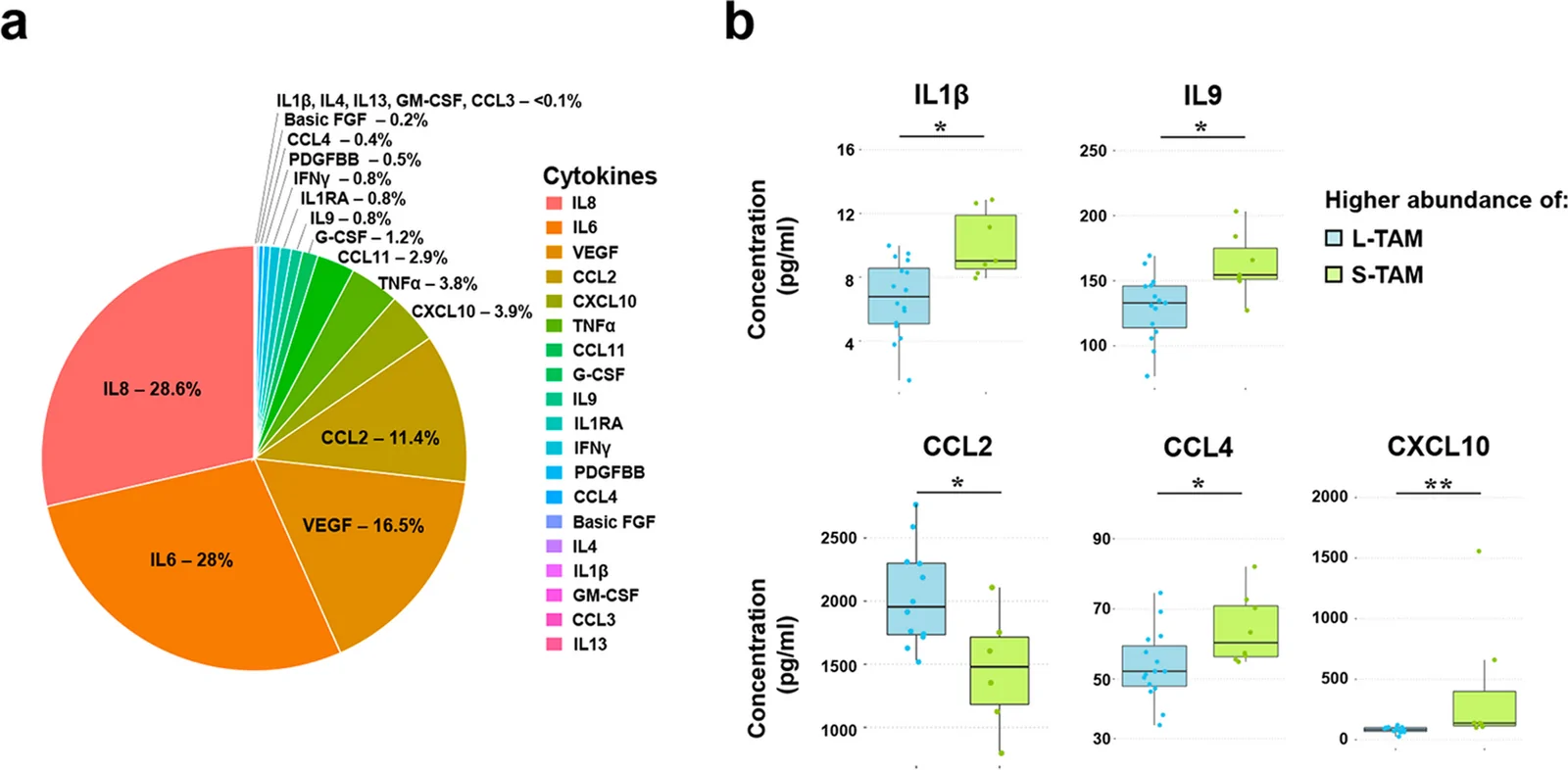
Profiling of cytokines released by patient-derived colorectal liver metastasis (CRLM) cells. **a** Mean cytokine concentration percentage (%) within the supernatants from primary CRLM cell cultures (*n* = 24) as determined using Bioplex. **b** Cytokine concentrations in patients stratified based on tumour-associated macrophage (TAM) morphology (small, S-TAM or large, L-TAM–enriched). Data is shown as mean ± SEM. *, *p* < 0.05, as compared with S-TAMs

We observed that tumour cells actively secrete a range of soluble factors that sustain cancer progression by enhancing their own proliferative capacity, establishing an immunosuppressive TME, and promoting angiogenesis and metastatic spread [2]. Among the most prominent factors identified were some interleukins (IL-6, IL-8), chemokines (CCL2, CCL4, CCL11, CXCL10), tumour necrosis factor-alpha (TNF-α), and vascular endothelial growth factor (VEGF) (Table S2; Fig. 4a).

To assess whether differences in tumour-derived soluble factors were associated with the distinct macrophage states observed in CRLM samples, patients were stratified according to macrophage size into S-TAM-enriched and L-TAM-enriched groups based on the predominant TAM morphology identified by IHC. Higher levels of IL1β, IL9, CXCL10, and CCL4 were detected in the supernatant of tumour cells with a high abundance of S-TAMs, reflecting more pronounced inflammatory features and an association with an M1-like phenotype while higher levels of CCL2 were observed in the supernatant of tumour cells with high abundance of L-TAMs (Fig. 4b).

### LSD1 inhibition reshapes TAM phenotype and counteracts tumour-induced macrophage reprogramming

**Fig. 5:**
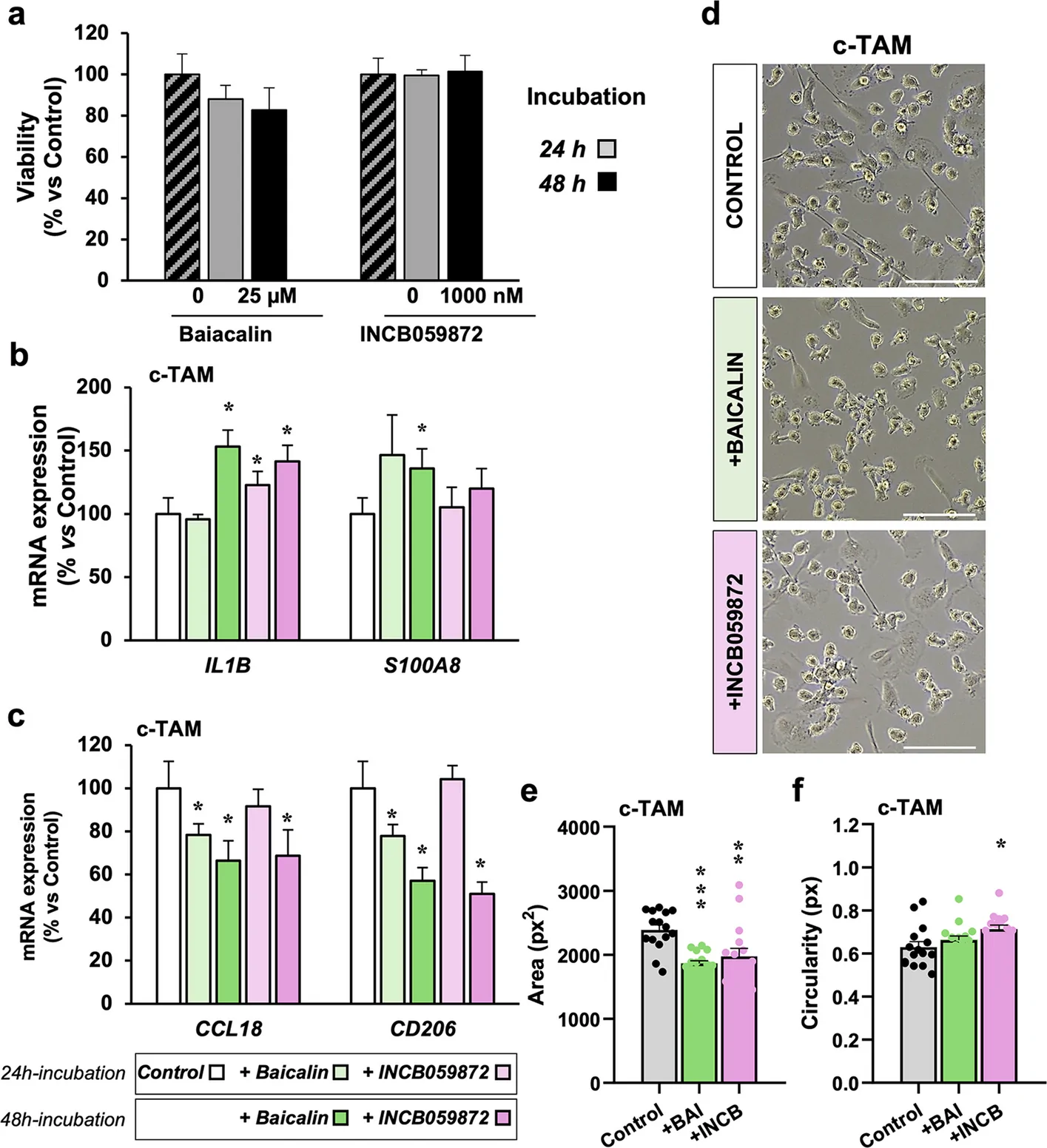
Pharmacological inhibition of LSD1 modulates macrophage functional state in in vitro-generated classical tumour-associated macrophages (c-TAMs). **a** Effect of selected concentrations of LSD1 inhibitors (baicalin or INCB059872) on the viability of monocyte-derived M0 macrophages. Expression of selected **b** S-TAM-associated and **c** L-TAM-associated genes in in vitro-generated TAMs incubated with medium alone (control) or with non-toxic concentrations of baicalin or INCB059872 for 24 or 48 h, as determined by RT-qPCR using *GAPDH* as an internal reference gene. **d** Bright-field microscopy images of cultured c-TAMs incubated for 24 h in the presence or absence (medium alone, control) of each LSD1 inhibitor (scale bar, 100 µm). Quantification of cell **e** area and **f** circularity of in vitro-generated TAMs incubated with medium alone (control) or with LSD1 inhibitors for 24 h using bright-field microscopy images (*n* = 15, with triplicates). Data are presented as mean ± SEM and expressed as a percentage relative to untreated controls. *, *p* < 0.05, **, *p* < 0.01, ***, *p* < 0.0001, as compared with untreated c-TAMs (Control)

We previously reported that L-TAMs exhibit higher levels of LSD1 [15]. To investigate whether LSD1 targeting could modulate TAM phenotypic features, we first determined non-toxic working concentrations for two LSD1 inhibitors (baicalin and INCB059872). Monocyte-derived M0 macrophages were treated for 24 and 48 h with increasing concentrations of each compound (µM range for baicalin and nM range for INCB059872) and cell viability was quantified to define the cytotoxic threshold. The concentrations selected for subsequent experiments (reported in Fig. S3b; Fig. 5a) corresponded to the highest doses that consistently preserved macrophage viability without detectable toxicity. In vitro-generated classical TAMs (c-TAMs) were then treated with the selected non-toxic concentrations and examined after 24 and 48 h to assess potential phenotypic changes (workflow in Fig. S3c). LSD1 inhibition induced morphological and transcriptional changes consistent with macrophage reprogramming towards a more inflammatory phenotype. LSD1 inhibition induced time-dependent transcriptional changes in TAM-associated programmes. c-TAMs treated with baicalin or INCB059872 up-regulated inflammatory macrophage-associated genes (*IL1B*, *S100A8*) at both 24 and, more prominently, at 48 h, while down-regulating genes associated with immunoregulatory/TAM-like states (*CCL18*, *CD206*) (Fig. 5b and c).

LSD1 inhibition was also associated with changes in macrophage morphology. The in vitro-generated c-TAMs shifted from an elongated, spiny phenotype to a smaller, rounded form after being treated for 24 h with both inhibitors (Fig. 5d). Quantitative analysis of cell morphology confirmed these observations, showing a significant reduction in cell area after 24 h of treatment with both compounds (Fig. 5e), together with increased circularity in macrophages treated with INCB059872 (Fig. 5f). These morphological changes were consistent with the transcriptional modulation observed following LSD1 inhibition.

**Fig. 6:**
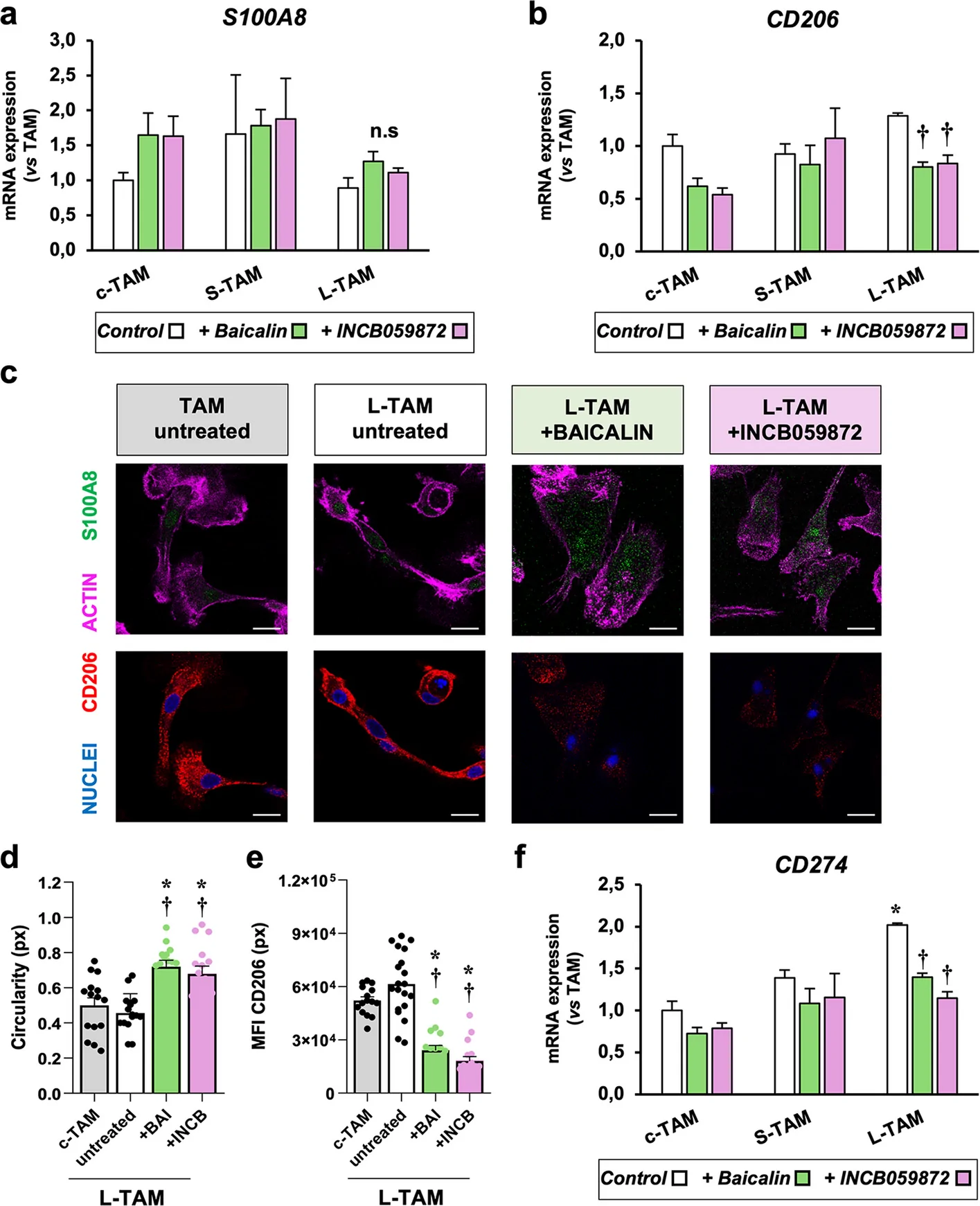
Modulation of the functional state of in vitro-generated TAM models derived from CRLM tumour-conditioned media by LSD1 inhibition. Expression of **a** *S100A8* and **b** *CD206* in in vitro*-* (classical) c-TAMs and TAM models generated using CRLM tumour-conditioned media from samples enriched in S-TAMs or L-TAMs incubated with medium alone (Control) or with non-toxic concentrations of LSD1 inhibitors (baicalin or INCB059872) for 48 h, as determined by RT-qPCR **c** Immunofluorescence staining of S100A8 (green) and actin filaments (with phalloidin in magenta) or CD206 (red) and nuclei (with Hoechst, blue) in in vitro-derived TAMs and L-TAMs, treated with either baicalin (BAI) or INCB059872 (INCB) (scale bar, 10 µm). Quantification of **d** cell circularity and **e** CD206 mean fluorescence intensity (MFI). **f** Expression of *CD274 *in vitro-generated c-TAMs and TAM models generated using CRLM tumour-conditioned media from samples enriched in S-TAMs or L-TAMs incubated with medium alone (Control) or with non-toxic concentrations of LSD1 inhibitors (baicalin or INCB059872) for 48 h, as determined by RT-qPCR. *GAPDH* and *18S* were used as normalizers. Data are expressed as folds relative to untreated classical TAMs (c-TAM) of each replicate. Data is shown as mean ± SEM in each case. *, *p* < 0.05, as compared with untreated classical c-TAMs; **†**, *p* < 0.05, as compared with the untreated corresponding TAM

To determine whether LSD1 inhibition could counteract tumour-derived signals responsible for establishing distinct TAM states, TAMs were subsequently exposed to tumour cell-derived supernatants from S-TAM-enriched or L-TAM-enriched CRLM samples and treated with LSD1 inhibitors for 48 h (Fig. 6). Macrophages exposed to L-TAM-derived conditioned media displayed increased *CD206* expression and a morphology associated with the L-TAM phenotype (Fig. 6c and e). Treatment with either baicalin or INCB059872 significantly reduced CD206 gene and protein expression (Fig. 6b and e), supporting the ability of LSD1 inhibition to modulate tumour-induced immunoregulatory macrophage programming. Morphological analysis further supported the phenotypic modulation induced by LSD1 inhibition. Both inhibitors promoted a shift towards a more rounded morphology, increasing cellular circularity compared with untreated L-TAM-like macrophages and conventional TAMs (Fig. 6d).

**Fig. 7:**
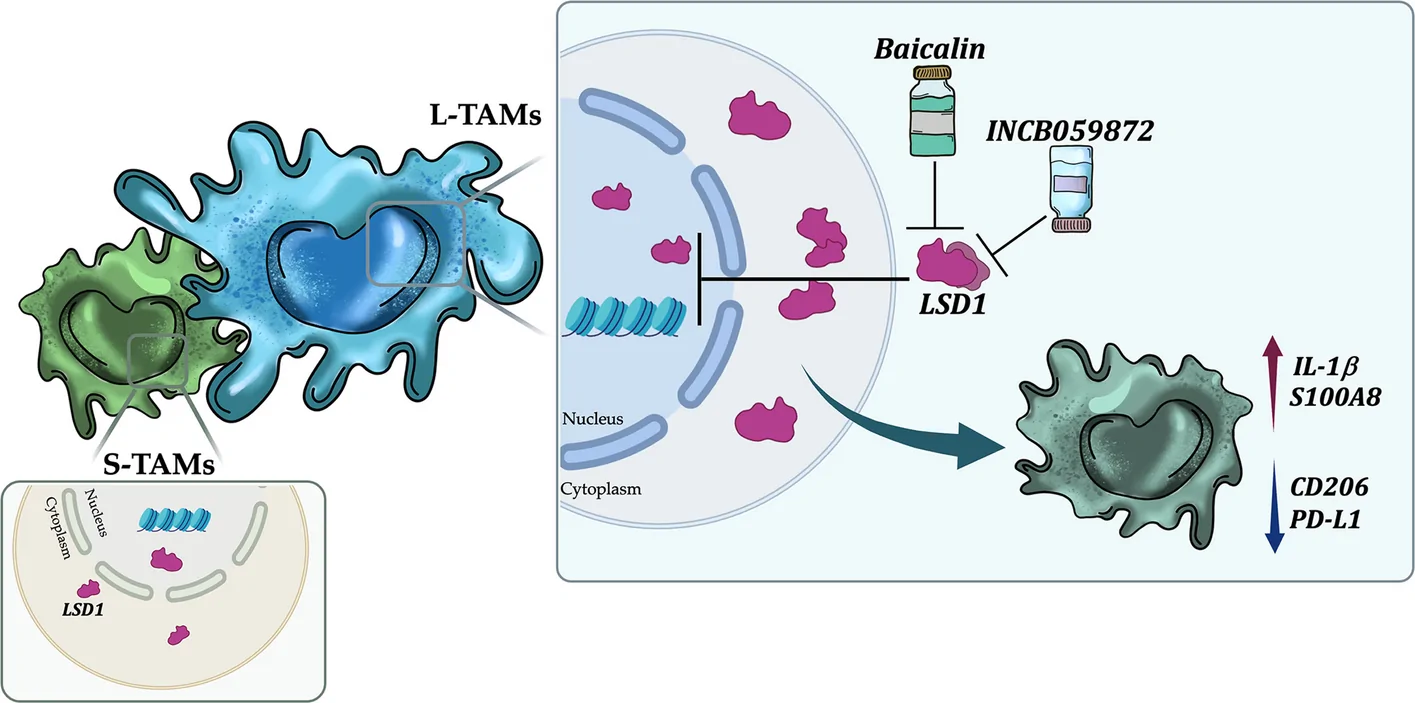
Summarising schematic illustrating the role of LSD1 in modulating tumour-associated macrophage (TAM) states in colorectal liver metastases (CRLM). LSD1 contributes to the maintenance of TAM transcriptional programmes. Pharmacological inhibition of LSD1 with baicalin or INCB059872 reduces selected immunoregulatory-associated features of TAMs, including CD206 and PD-L1 expression, supporting a role for LSD1 in regulating macrophage plasticity. Most illustrations were hand-drawn, whereas selected graphical elements were taken from BioRender.com. Large-TAM, L-TAMs; small-TAM, S-TAMs

Finally, to evaluate whether LSD1 inhibition also affects immune checkpoint-associated features, we analysed PD-L1 gene (*CD274*) expression. LSD1 inhibition decreased PD-L1 levels in TAMs, particularly in macrophages exposed to L-TAM-enriched media (Fig. 6f). Together, these results suggest that LSD1 inhibition limits tumour-induced immunoregulatory macrophage features by reducing CD206 and PD-L1 expression and promoting a less tumour-supportive macrophage phenotype (Fig. 7).

## Discussion

Tumour-induced immune remodelling in CRLM extends beyond the metastatic lesion, reshaping the macrophage landscape of the peritumoral liver into a distinct immunological niche enriched in tumour-conditioned, immunoregulatory populations, supporting the concept that the peritumoral compartment represents a specialised microenvironment for macrophage reprogramming rather than a passive tissue boundary [20, 21].

A notable observation is the marked accumulation of CD68‑high macrophages in peritumoral tissue. This homogeneous population shares key hallmarks with the L‑TAMs we previously described, including increased cell size and elevated CD163 expression [9]. In the present study, transcriptomic analysis further demonstrates that these morphologically defined macrophages correspond to distinct functional states, rather than representing a new taxonomic classification of TAMs. Accordingly, we use the S-TAM/L-TAM nomenclature as a morphology-based framework to distinguish macrophage states that are further supported by phenotypic and transcriptional features, rather than as a classification of distinct macrophage lineages or ontogenetic origins. These data support the concept that tumour-driven signals contribute to the establishment and maintenance of immunoregulatory macrophage states [22]. In contrast, CD68-dim macrophages are heterogeneous, encompassing a spectrum of functional states rather than discrete M1/M2 polarisation [23, 24]. The presence of HLA-DR-high macrophages suggests retained antigen-presenting or inflammatory potential although these populations are quantitatively overshadowed by immunoregulatory subsets, indicating a dominant shift in local macrophage programming rather than complete functional loss of inflammatory compartments. The transcriptomic characterisation further reinforces this interpretation. L-TAMs displayed enrichment of pathways related to tissue remodelling, receptor-mediated endocytosis, complement activation and angiogenesis, together with higher expression of immunoregulatory genes including MRC1, CD274, CCL18, VSIG4 and LYVE1. In contrast, S-TAMs were enriched for interleukin-1 signalling, leukocyte chemotaxis, T-cell activation and inflammatory response programmes, supporting their more inflammatory transcriptional profile. These findings place our morphologically defined TAM populations in the context of the broader landscape of functional macrophage states described by recent transcriptomic studies.

Together, these observations should be interpreted in the context of the complexity of hepatic macrophage biology: resident Kupffer cells and niche cues can be reprogrammed by metastases and may contribute to CD68 expression patterns and the phenotypic diversity observed [25]. Importantly, macrophage ontogeny and functional polarization should be considered as interconnected but not interchangeable concepts. While tissue-resident and monocyte-derived macrophages represent distinct developmental origins, the functional states acquired within the TME are primarily shaped by local environmental cues. Thus, L-TAMs and S-TAMs should be interpreted as morphologically defined functional states rather than surrogates of macrophage ontogeny. Their relationship may reflect macrophage plasticity and adaptation to local tumour-derived and tissue-derived cues, but whether these states represent sequential stages, alternative states, or arise from different cellular origins cannot be resolved in the present human CRLM cohort.

Supporting this interpretation, our data show that CRLM cells secrete cytokines that collectively shape TAM phenotypes and establish an immunosuppressive niche in the liver. High levels of CCL2, IL-6, IL-8 and VEGF found in CRLM tumour secretomes promote monocyte recruitment, survival signalling, M2-like polarisation, and angiogenesis, respectively [26–31] suggesting that tumour-derived soluble factors act as upstream drivers of macrophage state modulation rather than isolated signalling events thereby contributing to therapy resistance in liver cancer [32, 33]. These interactions create a self-reinforcing loop whereby tumour cells educate infiltrating myeloid cells into tolerogenic TAMs, which in turn release IL-6, VEGF, matrix proteases, and immune checkpoints that enhance metastatic seeding, stromal remodelling, and T-cell suppression [34]. Notably, CCL2 is enriched in tumours associated with L-TAM predominance, consistent with enhanced recruitment of immunosuppressive macrophages [35]. In contrast, tumours enriched in S-TAMs display a distinct cytokine profile, characterised by higher levels of IL-1β, CXCL10, CCL4, and IL-9, associated with inflammatory signalling and anti-tumour immunity [36–40]. These data support a model in which tumour-derived cytokine gradients drive macrophage heterogeneity and functional polarisation and highlight the tumour margin as a dynamic hub for tumour-immune crosstalk [41]. Accordingly, an immunosuppressive, M2-skewed TAM phenotype in the metastatic niche is associated with therapy resistance and poor prognosis [42], as reflected by the association between L-TAM enrichment and early recurrence in our cohort [9, 19]. The S-TAM/L-TAM framework provides a practical morphology-based system for describing this functional heterogeneity, linking readily identifiable cellular features with distinct inflammatory or immunoregulatory programmes and clinical outcome. Importantly, the ability of tumour-derived conditioned media to induce partial L-TAM-like or S-TAM-like features in vitro does not imply that tumour-derived factors alone define TAM identity. Rather, these findings support a model in which tumour-secreted mediators act together with local tissue-derived signals to shape macrophage functional states within the metastatic niche.

In this context, epigenetic regulation emerges as a key mechanism controlling macrophage identity and function. LSD1 acts as a central regulator of macrophage plasticity, linking chromatin remodelling to immune suppression [43]. Pharmacological inhibition of LSD1 using baicalin or INCB059872 [44] attenuates selected immunoregulatory features of TAMs, while preserving most inflammatory-associated gene expression, suggesting that LSD1 contributes predominantly to the maintenance of immunoregulatory transcriptional programmes rather than acting as a global regulator of macrophage activation. This is relevant to the L-TAM phenotype, which is characterised by an immunoregulatory transcriptional programme. The modulation of selected immunoregulatory features following LSD1 inhibition suggests that epigenetic mechanisms may contribute to the regulation of macrophage functional states. These findings are consistent with previous reports demonstrating that LSD1 inhibition can modulate macrophage-associated programmes and reduce markers associated with immunoregulatory macrophage states [45]. The delayed effect observed after LSD1 inhibition is consistent with the time-dependent nature of epigenetic reprogramming, where changes in chromatin accessibility and transcriptional networks precede detectable modulation of macrophage-associated markers.

The reduction of PD-L1 expression identifies LSD1 as a regulator of immune checkpoint expression in macrophages. Consistent with the role of macrophage-derived PD-L1 in limiting cytotoxic T-cell responses, these findings suggest that LSD1 inhibition may partially alleviate macrophage-mediated immunosuppression [46, 47]. However, whether this translates into enhanced T-cell function or improved responses to immune checkpoint blockade remains to be established.

From a translational perspective, our findings identify LSD1 as a promising target to modulate macrophage function within the metastatic microenvironment [48]. Although the therapeutic benefit of combining LSD1 inhibition with PD-1/PD-L1 blockade has not yet been established in CRLM, preclinical studies in other tumour types have shown that such combinations can reduce immunosuppressive cytokine production and partially restore T-cell function [49] and early-phase clinical trials are currently exploring these combinations in patients with small-cell lung cancer (NCT06287775), supporting the broader relevance of epigenetic-myeloid targeting across tumour types. In particular, our results provide a rationale for future studies exploring this strategy in human models of CRLM, including more complex multicellular systems capable of recapitulating tumour-immune cell interactions.

While our study provides a comprehensive molecular and functional characterisation of morphologically distinct TAM populations in human CRLM, it also opens several avenues for future investigation. The present work was designed to define the transcriptional and phenotypic consequences of LSD1 inhibition on human macrophages, whereas its impact on macrophage spatial organisation within the metastatic niche and the ontogeny or potential interconversion of S-TAM and L-TAM states remains to be explored. Lineage-tracing or longitudinal approaches would be required to determine their developmental relationship.

Several limitations of the present study should be acknowledged. First, although the observed reduction in PD-L1 expression suggests that LSD1 inhibition may alleviate macrophage-mediated immunosuppression, its consequences for T-cell activation and anti-tumour immunity will require validation in more complex experimental systems. Patient-derived organoids, multicellular co-culture models incorporating immune cells, in vivo or lineage-resolved approaches will provide valuable platforms to address these questions. Nevertheless, the use of primary human CRLM specimens combined with complementary in vitro models provides a robust and translationally relevant framework for defining macrophage regulatory programmes and their epigenetic modulation and identifies LSD1 as a promising regulator of macrophage plasticity in CRLM.

## Conclusion

In summary, our findings demonstrate that morphologically distinct TAMs represent functionally divergent macrophage states within the peritumoral niche of CRLM. L-TAMs display a transcriptional and phenotypic programme consistent with an immunoregulatory state and are associated with poor clinical outcome, whereas S-TAMs exhibit a more inflammatory profile. Pharmacological inhibition of LSD1 attenuates selected immunoregulatory features of macrophages, including CD206 and PD-L1 expression, supporting a role for LSD1 in maintaining macrophage plasticity. Together, these findings position LSD1 as a promising epigenetic regulator of the metastatic immune microenvironment, and provide a rationale for future studies exploring its therapeutic potential, alone or in combination with immunomodulatory strategies.

## Materials and methods

### Study population

The study was approved by the Institutional Ethics Committee (approval no. 1341) and conformed to the ethical guidelines of the Declaration of Helsinki. Informed consent was obtained from all subjects involved in the study. Human liver samples were collected from patients undergoing liver resection for CRLM at IRCCS Humanitas Research Hospital (Milan, Italy) between 2020–2024. The inclusion criteria were: 1) pathology-proven CRLM; 2) age ≥ 18 years old. Exclusion criteria were: 1) follow-up < 6 months, unless an earlier death event occurred. For each patient, paired samples from healthy liver tissue (> 2 cm from tumour margin) and peritumoral tissue (< 1 cm from tumour margin) were obtained from the surgical specimen. Samples were processed within 2 h of excision. Resected CRLM patients were followed up every 3 months, as per protocol in the enrolling centre, or until death, and major events were recorded. Baseline characteristics of the cohort are summarized in Table 1 and Table S1.

### Cell culture of primary tumour cells

The tumoral tissue of the patients was manually minced and enzymatically digested. Enzymatic digestion was carried out by incubating the fragmented tissues in Hanks’ Balanced Salt Solution (HBSS) (Euroclone) supplemented with 100 U/ml Type I Collagenase (Gibco). The tubes were then placed at 37 °C on a rotating platform for 45 min, allowing tissue digestion. The sample was filtered through a 100 μm nylon cell strainer to obtain a uniform single-cell suspension and centrifuged at 2000 RPM for 5 min to pellet the isolated cells. After centrifugation, the supernatant was carefully discarded, and the cell pellet was washed twice with PBS 1x-/- (Sigma-Aldrich), to eliminate any remaining tissue. Cells were counted and cultured in RPMI 1640 (Euroclone), supplemented with 20% heat-inactivated foetal bovine serum (FBS) (Euroclone), 1% nonessential amino acids (NEAA, Lonza), 1% glutamine (Lonza), and 1% penicillin/streptomycin (Sigma-Aldrich) in a 37 °C, 5% CO_2_ incubator. Conditioned medium was obtained by collecting the culture supernatant from 25 primary tumour cells after 48 h of confluence, followed by centrifugation at 800 g for 10 min. The clarified supernatant was then stored at −80 °C until use.

### Sorting of macrophages

Macrophage subsets (S- and L-TAMs) were sorted using a BD FACSAria™ III (BD Biosciences) system from the peritumoral CRLM specimens (*n* = 5). Single-cell suspension was obtained by tissue digestion as described in the previous section. Then, cells were incubated with a blocking solution (saline solution with 1% human serum) and stained with the following fluorophore-conjugated primary antibodies: anti-CD45 (BD Biosciences; clone HI30 RRID:AB_1645480), anti-CD11b (BD Biosciences; clone ICRF44 RRID:AB_398456), anti-CD16 (BioLegend; clone 3G8 RRID:AB_2563639), anti-CD14 (BD Biosciences; clone M5E2 RRID:AB_2744286), anti-CD66b (BioLegend; clone G10F5 RRID:AB_2566605), and anti-CD163 (BD Biosciences; clone GHI/61 RRID:AB_2033943). Fixable Viability Stain 700 fluorescent dye (BD Biosciences RRID: AB_2869637) was used for dead cell exclusion. SYTO 16 Green Fluorescent Nucleic Acid Stain (Invitrogen RRID: AB_2890138) was used to identify nucleated cells. 100,000 cells were FACS sorted for each type (small or large-TAMs) following the gating strategy as previously published [9].

The freshly sorted L- and S-TAMs were stored at −80 °C until being subjected to RNA extraction and from one patient were processed for electron microscopy imaging (see sections below).

### Macrophage differentiation from monocytes and cell culture

Peripheral blood mononuclear cells (PBMCs) were isolated from buffy coats of healthy donors by Ficoll-Paque density gradient centrifugation. CD14⁺ monocytes were enriched using Plurispin (Pluriselect) and cultured in RPMI 1640 supplemented with 10% heat-inactivated FBS, 1% penicillin/streptomycin, and recombinant human M-CSF (50 ng/mL, #300–25, PeproTech) for 7 days to induce macrophage differentiation. To create conditioned macrophages, monocytes were cultured in a 1:1 (v:v) ratio of conditioned medium and complete RPMI plus the addition of IL-4 (1 μg/mL, # 200–04, PeproTech), IL-10 (1 μg/mL, # 200–10, PeproTech), and M-CSF (1 μg/mL, #300–25, PeproTech) and cells were harvested on day 7 as reported by Benner et al. [50]. Medium and cytokines were refreshed every other day. To evaluate the effects of LSD1 inhibition, in vitro-generated TAMs were incubated with non-toxic concentrations of baicalin (25 µM) or INCB059872 (1000 nM) for 24 or 48 h while cells incubated with medium alone served as control.

### Characterisation of macrophages from patients with CRLM by flow cytometry

Fresh liver tissue (healthy and peritumoral) was mechanically minced and enzymatically digested as described before. Cell suspensions were filtered through a 100 µm strainer and red blood cells were lysed with BD lysis buffer (BD Biosciences Cat# 349,202, RRID:AB_2868862). Cells were stained with these antibodies CD15 BV711 (BD Biosciences RRID:AB_2917402), HLA-DR BV510 (BD Biosciences RRID:AB_2870495), CD206 BV421 (BD Biosciences RRID:AB_2869936), CD123 PE-CF594 (BD Biosciences RRID:AB_11153664), CD11b BV605 (BD Biosciences RRID:AB_2737745), CD163 Alexa647 (BD Biosciences RRID:AB_2737710), CD16 BV786 (BD Biosciences RRID:AB_2744299), CD14 BV650 (BD Biosciences RRID:AB_2744286), CD33 PE-Cy7 (BD Biosciences RRID:AB_2713932), CD45 APC-H7 (BD Biosciences RRID:AB_1645480). After fixation and permeabilisation the cells were incubated with CD68 PE (BD Biosciences RRID:AB_396345) and Syto16 (Invitrogen RRID: AB_2890138). Data were acquired on a BD LSRFortessa™ and analised with FlowJo v10. Gating strategy excluded debris through Syto16 staining and doublets, selected CD45⁺ immune cells, identified myeloid cells as CD33^+^ and particularly macrophages as CD15-CD123-CD11b⁺CD68⁺. UMAP dimensionality reduction was employed to visualize macrophage subsets.

#### Quantitative PCR

RNA was extracted using the RNeasy Mini Kit (#74,134, Qiagen). cDNA was synthesized with High-Capacity Reverse Transcription Kit (#4,368,814, Applied Biosystems). Quantitative PCR was performed with SYBR Green Master Mix (#4,309,155, Applied Biosystems) on a QuantStudio 5 system. Relative gene expression was calculated by the ΔCt method, normalized to *GAPDH* alone or double normalized in combination with *18S*. Primers targeted M1 (e.g., IL1β, S100A8), M2 (e.g., CCL18, CD206), and other genes related to immune response (e.g. CD274). Primer sequences are listed in Supplementary Table S3.

#### Cell viability assays

Cell viability was assessed using the RealTime-Glo® Viability Assay (#G9711, Promega) at endpoint. Briefly, monocytes were seeded in 96-well plates at a density of 4 × 10^3^ cells per well and differentiated into macrophages with M-CSF (50 ng/mL, #300–25, PeproTech) for 7 days. Differentiated macrophages were then exposed to increasing concentrations of the LSD1 inhibitors baicalin or INCB059872 for 24 or 48 h. Luminescence was measured using a GloMax® Discover microplate reader (Promega) according to the manufacturer’s instructions. Viability values were normalized to untreated controls.

#### Immunohistochemistry (IHC)

Paraffin-embedded CLM tissue was provided by the Pathology Department of the Humanitas Clinical and Research Hospital. After section deparaffinisation and rehydration, antigen retrieval was performed using DIVA buffer (Biocare) in a decloaking chamber (Biocare). Endogenous peroxidases were blocked with Peroxidase-Blocking Solution (Dako) for 15 min at room temperature (RT). The sections were then incubated with anti-human CD163 (1:600, Leica Biosystems RRID:AB_2756375), overnight at 4 °C. EnVision + System HRP-conjugated secondary antibody (Dako) was applied for 30 min and Betazoid DAB (Biocare) was used as the chromogen. Nuclei were counterstained with Dako REAL hematoxylin (Dako).

#### Immunofluorescence (IF)

Immunofluorescent staining was carried out on cells grown on coverslips, which were fixed with 4% formalin (10 min) and permeabilized with 0.5% Triton X (60 min, RT). Then, after washes the cells were incubated with antibodies against S100A8 (F4R2W) (Cell Signaling Technology Cat# 47,310, RRID:AB_3698766) and CD206 (R&D Systems Cat# MAB25341, RRID:AB_10890782), diluted to 1:50. After washes with PBS the cells were incubated with the secondary antibodies, anti-rabbit Alexa Fluor-488 (Thermo Fisher Scientific, Cat# SA5-10,383-AFP488, RRID:AB_3719791) and anti-mouse Alexa Fluor-Texas Red (Thermo Fisher Scientific Cat# T-862, RRID:AB_2556781) diluted to 1:500 and with Alexa Fluor™ 647 phalloidin (1:200, Thermo Fisher Scientific Cat# A22287, RRID:AB_2620155). Nuclei were counterstained with Hoechst 33,342 (diluted 1:5000, Cell Signaling Technology Cat# 4082, RRID:AB_10626776). The images were acquired by Leica TCS SP2 confocal microscope. Image analysis and quantification were performed using ImageJ software (National Institutes of Health, Bethesda, MD, USA).

### TEM electron microscopy

The macrophages of one patient were sorted in S- and L-TAMs as previously described and fixed in fixative solution (2% glutaraldehyde in Sodium Cacodilate Buffer 0.1 M pH 7.4) for 1 h at RT. Cells were then washed with the buffer and post-fixed in aqueous osmium tetroxide solution (1%). Next, the specimens were dehydrated through a series of ethanol solutions (50%−70%−90%−95%−100%), incubated in grated absolute ethanol/propylene oxide solutions and then in grated propylene oxide/epoxy resin mixtures. Specimens were placed into a fresh change of pure resin for at least 4 h, then embedded in another fresh change of 100% resin, and placed in a 60 °C oven at least for 48 h for polymerisation. Samples were cut with an ultramicrotome (Reichert) and ultrathin Sects. (50–70 nm) were double stained with uranyl acetate and lead citrate solutions. TEM images were taken with JEOL JEM-2100 Plus (JEOL, Italy) transmission electron microscope at 80 kV.

### Cytokine profiling

Cytokine concentrations were measured in the supernatants from several primary tumour cell cultures (*n* = 24)—obtained from patients who underwent surgical resection of CRLM—using the Bio-Plex Pro™ Human Cytokine Panel (##M500KCAF0Y, Bio-Rad), according to the manufacturer’s protocol. Data were acquired on a Bio-Plex 200 system and analysed with Bio-Plex Manager software.

### Differential gene expression and pathway enrichment analysis of S-TAMs and L-TAMs

Previously generated bulk RNA-sequencing data from paired S-TAM and L-TAM peritumoral macrophage populations from human CRLM (*n* = 5) were obtained from GEO accession GSE131353. TMM-normalised expression values were log2-transformed, and differential expression between L-TAMs and S-TAMs was assessed using the limma package, with patient pair and macrophage group included in the design matrix. S-TAMs were used as the reference group, such that positive log2 fold changes indicated higher expression in L-TAMs. Genes were ranked by their moderated t-statistic and analysed by pre-ranked Gene Set Enrichment Analysis (GSEA) using the fgsea package and Gene Ontology Biological Process gene sets from the Molecular Signatures Database (MSigDB) containing between 15 and 500 genes. Pathways with a Benjamini–Hochberg false discovery rate (FDR) below 0.05 were considered significantly enriched, with enrichment direction determined by the sign of the normalized enrichment score (NES). A set of representative differentially expressed genes was selected to define L-TAM and S-TAM transcriptional signatures and displayed as row-wise Z-scores of log2-transformed TMM-normalized expression values.

### Statistical analysis

Clinical categorical variables were reported as frequencies and percentages, while continuous variables were expressed as medians with their respective interquartile ranges (IQR). Experimental data are presented as mean ± SEM unless otherwise specified. Comparisons were performed using Mann–Whitney U, Chi-Square or Fisher’s, and unpaired Student’s t-test, as appropriate. Multiple group comparisons were performed by one-way ANOVA with Tukey’s post-hoc test. Overall Survival (OS) and RFS analyses were conducted using the Kaplan–Meier method, with group comparisons performed via the log-rank test. All statistical tests were two-sided, and a *p*-value < 0.05 was considered statistically significant. The statistical analyses were performed using GraphPad (v10) and R Statistical Software (v4.5.1).

## Supplementary Information

Supplementary Material 1.

## Data Availability

The data supporting the findings of this study are available from the corresponding author upon reasonable request.
