# Supplementary material for: Lysine-specific demethylase 1 (LSD1) regulates macrophage plasticity and immune checkpoint expression in colorectal liver metastases
Source: Mol Biomed. 2026 Oct 20;7:219. doi: 10.1186/s43556-026-00602-5 (PMC13683283; doi:10.1186/s43556-026-00602-5)
Supplement: Supplementary file 1 [file 43556_2026_602_MOESM1_ESM.docx]

**Journal:** Molecular Biomedicine

**Supplementary material**

**Title:** Lysine-specific demethylase 1 (LSD1) Regulates Macrophage Plasticity and Immune Checkpoint Expression in Colorectal Liver Metastases

**Authors:**

Candela Cives-Losada^1,2^*^#^*, Cristiana Soldani^2^*^#^*, Michela Anna Polidoro^2,3^, Barbara Franceschini^2^, Flavio Milana^3,4^, Aurora Gazzillo^2^, Antonella Cammarota^2^, Chiara Camisaschi^5^, Francesca Clemente^6^, Achille Anselmo^6^, Luca Di Tommaso^3,7^, Guido Torzilli^3,4^, Domenico Mavilio^8,9^, Ana Lleo^3,10^, Matteo Donadon^1,11*^

**Affiliations:**

^1^ Department of Health Sciences, Università del Piemonte Orientale, Novara, Italy

^2^ Laboratory of Hepatobiliary Immunopathology, IRCCS Humanitas Research Hospital, Rozzano, Milan, Italy

^3^ Department of Biomedical Sciences, Humanitas University, Pieve Emanuele, Milan, Italy

^4^ Division of Hepatobiliary and General Surgery, Department of Surgery, IRCCS Humanitas Research Hospital, Rozzano, Milan, Italy

^5^ Flow Cytometry Core, IRCCS Humanitas Research Hospital, Rozzano, Milan, Italy

^6^ Spectral flow Unit, Fondazione Istituto Nazionale di Genetica Molecolare-INGM, Milan, Italy

^7^ Department of Pathology, IRCCS Humanitas Research Hospital, Rozzano, Milan, Italy

^8^ Unit of Clinical and Experimental Immunology, IRCCS Humanitas Research Hospital, Rozzano, Milan, Italy

^9^ Department of Medical Biotechnology and Translational Medicine, University of Milan, Milan, Italy

^10^ Division of Internal Medicine and Hepatology, Department of Gastroenterology, IRCCS Humanitas Research Hospital, Rozzano, Milan, Italy

^11^ Division of General Surgery, University Maggiore Hospital della Carità, Novara, Italy

^#^ These authors contributed equally as co-first authors.

^*^ Corresponding author.

**Corresponding author:**

Matteo Donadon, MD, PhD

E-mail: matteo.donadon@uniupo.it

ORCID: 0000-0003-0296-7648

**Supplementary Table S1.** Demographic and clinical characteristics of the CRLM cohort analysed (n = 33).

| **VARIABLE** | **N=33** |
| --- | --- |
| **Macrophage size, large,** n (%) | 17 (51.5) |
| **Age,** (Median [IQR]) | 59.90 [49.88, 71.96] |
| **Sex, male,** n (%) | 21 (63.6) |
| **BMI** (Median [IQR]) | 24.44 [21.61, 26.45] |
| **Site of primary tumor,** n (%) |  |
| Right | 8 (24.2) |
| Transverse | 3 (9.1) |
| Left | 11 (33.3) |
| Rectum | 11 (33.3) |
| **Grade stage 3,** n (%) | 7 (33.3) |
| **Tumor stage,** n (%) |  |
| 1-2 | 3 (9.0) |
| 3-4 | 30 (91.0) |
| **Nodal stage,** n (%) |  |
| 0 | 5 (15.2) |
| 1 | 16 (48.5) |
| 2 | 12 (36.4) |
| **Neoadjuvant CHT, yes,** n (%) | 30 (90.9) |
| **Response** to CHT  CR  PR  SD  PD | 0/30 (0.0)  23/30 (76.7)  5/30 (16.7)  2/30 (6.6) |
| **Extrahepatic disease, yes,** n (%) | 5 (15.2) |
| **Synchronous disease, yes,** n (%) | 31 (93.9) |
| **Bilobar disease, yes,** n (%) | 26 (78.8) |
| **BRAF mutated, yes,** n (%) | 0 (0) |
| **KRAS mutated, yes,** n (%) | 10 (30.3) |
| **CEA** (Median [IQR]) | 4.10 [3.00, 8.90] |
| **CA19-9** (Median [IQR]) | 13.50 [4.80, 34.20] |
| **Number of tumor** (Median [IQR]) | 5.00 [3.00, 10.00] |
| **Size of largest tumor** (Median [IQR]) | 3.20 [2.10, 4.10] |
| **Adjuvant CHT, yes,** n (%) | 21 (63.6) |

*BMI: body-mass index; CHT: chemotherapy; CR: complete response; PR: partial response; SD: stable disease; PD: progressive disease; BRAF: B-Raf proto-oncogene; KRAS: Kirsten rat sarcoma virus oncogene homolog; CEA: carcinoembryonic antigen.*

**Supplementary Table S2.** Cytokine concentration in the supernatants from several primary tumour cell cultures obtained from patients who underwent surgical resection of CRLM (n = 24).

| **Cytokine**  **(name)** | **Concentration (pg/ml)** |  | **Cytokine**  **(name)** | **Concentration (pg/ml)** |  | **Cytokine**  **(name)** | **Concentration (pg/ml)** |
| --- | --- | --- | --- | --- | --- | --- | --- |
| **Basic FGF** | 25.1 ± 1.51 |  | **IFNγ** | 120 ± 4.6 |  | **IL4** | 14.1 ± 1.1 |
| **CCL2** | 1773 ± 105 |  | **IL10** | 1.74 ± 0.22 |  | **IL5** | 267.1 ± 13.0 |
| **CCL3** | 1.76 ± 0.42 |  | **IL12p70** | 1.23 ± 0.37 |  | **IL6** | 4371 ± 326 |
| **CCL4** | 55.5 ± 2.9 |  | **IL13** | 1.44 ± 0.19 |  | **IL7** | 51.8 ± 5.8 |
| **CCL5** | 25.4 ± 2.7 |  | **IL15** | 90.4 ± 9.5 |  | **IL8** | 4467 ± 1025 |
| **CCL11** | 448 ± 139 |  | **IL17** | 19.3 ± 1.8 |  | **IL9** | 132.4 ± 7.8 |
| **CXCL10** | 615 ± 358 |  | **IL1RA** | 130 ± 60.2 |  | **PDGFBB** | 78.4 ± 5.4 |
| **G-CSF** | 185 ± 111 |  | **IL1β** | 7.50 ± 0.60 |  | **TNFα** | 586 ± 35 |
| **GM-CSF** | 4.69 ± 1.33 |  | **IL2** | 4.65 ± 0.47 |  | **VEGF** | 2581 ± 499 |

Values are Mean ± SEM of duplicate determinations.

**Supplementary Table S3.** Forward (F) and reverse (R) primers used to determine the genes expression levels by RT-qPCR.

| **Gene** | **Protein** | **Primers** | **Type** | **Amplicon (bp)** | **Accesion number** |
| --- | --- | --- | --- | --- | --- |
| ***18S*** | - | CGCCGCTAGAGGTGAAATTC | F | 52 | NR_145820.1 |
|  |  | CTTTCGCTCTGGTCCGTCTT | R |  |  |
| ***GAPDH*** | GAPDH | CAGTCAGCCGCATCTTCTTT | F | 80 | NM_002046.7 |
|  |  | TTGACTCCGACCTTCACCTT | R |  |  |
| ***IL1B*** | IL1β | AGTCTGCCCAGTTCCCCAAC | F | 94 | NM_000576.3 |
|  |  | GTTATATCCTGGCCGCCTTT | R |  |  |
| ***S100A8*** | S100A8 | GGGCATCATGTTGACCGAGC | F | 298 | NM_002964.5 |
|  |  | GTAACTCAGCTACTCTTTGTGGC | R |  |  |
| ***CCL18*** | CCL18 | CCCAGCTCACTCTGACCACT | F | 160 | NM_002988.4 |
|  |  | GTGGAATCTGCCAGGAGGTA | R |  |  |
| ***CD206*** | CD206 | ATGCTCTACAAGGGATCGGG | F | 139 | NM_002438.4 |
|  |  | ACTTGAACGGGAATGCACAG | R |  |  |
| ***CD274*** | PDL1 | GGAACGGGACAGTATTTATG | F | 150 | NM_014143.4 |
|  |  | AAGCAGCAAGTTTAGTTTGG | R |  |  |

bp, base pairs; F, forward; R, reverse.

**Supplementary Figures**

**Supplementary Fig. S1 Flow cytometry analysis of human mono/macrophage cells from colorectal liver metastasis (CRLM).**  Doublets and debris were excluded based on pulse geometry and DNA content (using DNA intercalating agent Syto16, FITC-A), while dead cells were excluded using an amine-reactive viability dye (Live/Dead, FSV APC-R700-A). The representative gating strategy illustrates the identification of mono/macrophages defined as CD45^+^/CD33^+^/CD123^-^/CD15^-^/CD11b^+^/CD68^+^ from either healthy or peritumoral tissues.

**Supplementary Fig. S2 Immunophenotyping of mono/macrophages clusters identified through unsupervised data analysis of colorectal liver metastasis (CRLM).** **a** Histograms showing the relative expression of the following markers: CD68, CD14, CD206, CD16, CD163 and HLA-DR in the seven clusters corresponding to mono/macrophages identified through unsupervised analysis. **b and c,** Representative comparative analysis of CD163, CD206, HLA-DR and forward scatter (FSC) signals among CRLM-peritumoral mono/macrophages with high (hi) vs low (dim) levels of CD68. **d** Validation of Cluster 1, 2, 3 and 4 performed through manual gate; representative scatter plots are shown.

**Supplementary Fig. S3 Applied methodologies. a** Schematic representation of the methodology used to generate *in vitro* models of tumour-associated macrophages (TAMs) from patients with colorectal liver metastases (CRLM), in which monocyte-derived macrophages were exposed to conditioned medium from CRLM primary tumour cells. **b** Effects of LSD1 inhibitors on monocyte-derived M0 macrophage viability after 24- and 48-hour incubation with increasing concentrations of baicalin or INCB059872. **c** Experimental timeline illustrating the treatment of *in vitro*-generated TAMs with LSD1 inhibitors. Illustrations were hand-drawn by the authors.
